# New Insights in *Candida albicans* Innate Immunity at the Mucosa: Toxins, Epithelium, Metabolism, and Beyond

**DOI:** 10.3389/fcimb.2020.00081

**Published:** 2020-03-03

**Authors:** Aize Pellon, Shervin Dokht Sadeghi Nasab, David L. Moyes

**Affiliations:** Centre for Host-Microbiome Interactions, Faculty of Dentistry, Oral and Craniofacial Sciences, King's College London, London, United Kingdom

**Keywords:** epithelial cells, *Candida albicans*, candidalysin, immunometabolism, innate immune memory, trained immunity, IL-36 cytokine family

## Abstract

The mucosal surfaces of the human body are challenged by millions of microbes on a daily basis. Co-evolution with these microbes has led to the development of plastic mechanisms in both host and microorganisms that regulate the balance between preserving beneficial microbes and clearing pathogens. *Candida albicans* is a fungal pathobiont present in most healthy individuals that, under certain circumstances, can become pathogenic and cause everything from mild mucosal infections to life-threatening systemic diseases. As an essential part of the innate immunity in mucosae, epithelial cells elaborate complex immune responses that discriminate between commensal and pathogenic microbes, including *C. albicans*. Recently, several significant advances have been made identifying new pieces in the puzzle of host-microbe interactions. This review will summarize these advances in the context of our current knowledge of anti-*Candida* mucosal immunity, and their impact on epithelial immune responses to this fungal pathogen.

## Introduction

Humans host a myriad of different microbial species that inhabit the various body surfaces. This includes fungal species such as *Candida* spp., a major component of the mycobiome of ~70% of the healthy population (Schulze and Sonnenborn, 2009; Witherden et al., 2017). However, these species are pathobionts, capable of becoming pathogenic when local environmental conditions change (e.g., dampened host immunity, dysbiotic microbiota, changing pH/nutrients). Of these species, *C. albicans* is the most relevant, causing the majority of both colonization and infection events. In otherwise healthy patients, this fungus causes mild superficial mucosal infections with significant morbidity, such as oral thrush and vulvovaginal candidiasis (highly prevalent and recurrent in women). Whilst not associated with a high mortality, these superficial *C. albicans* infections can lead to systemic candidiasis in patients subjected to complex medical procedures, such as use of catheters, gut surgery or liver transplantation. In fact, *Candida* disseminated infections are the fourth most common nosocomial bloodstream infections, and have high associated mortality of 45–75% (Brown et al., 2012). In this context, both the host and *C. albicans* have co-evolved and developed mechanisms facilitating the adaptation.

Maintaining the proper balance and kinetics of immune responses at mucosal surfaces is critical for preserving homeostasis maintenance and commensal microbial communities whilst successfully clearing pathogens. Recently, epithelial cells have been identified as key players in these processes and not just mere physical barriers blocking the entrance of invading microorganisms, such as *C. albicans*. They are able to develop elaborate immune responses that discriminate between commensal and pathogenic microbes, and contribute to delaying and dampening infections, initiating the development of an immune response, as well as attracting immune cells to the infectious foci. Most recently, there has been a series of paradigm shifting discoveries that have had a dramatic impact on our understanding of the events that occur at epithelial surfaces during *C. albicans* infections, leading to several new potential routes for therapeutic intervention.

In this review we will discuss these recent findings regarding innate immunity to *C. albicans* in mucosal surfaces, with special emphasis on new insights and hypothesis that are changing our understanding of fungal-host interactions at the mucosae.

## Anti-*Candida* Innate Immunity at the Mucosa

**Figure 1:**
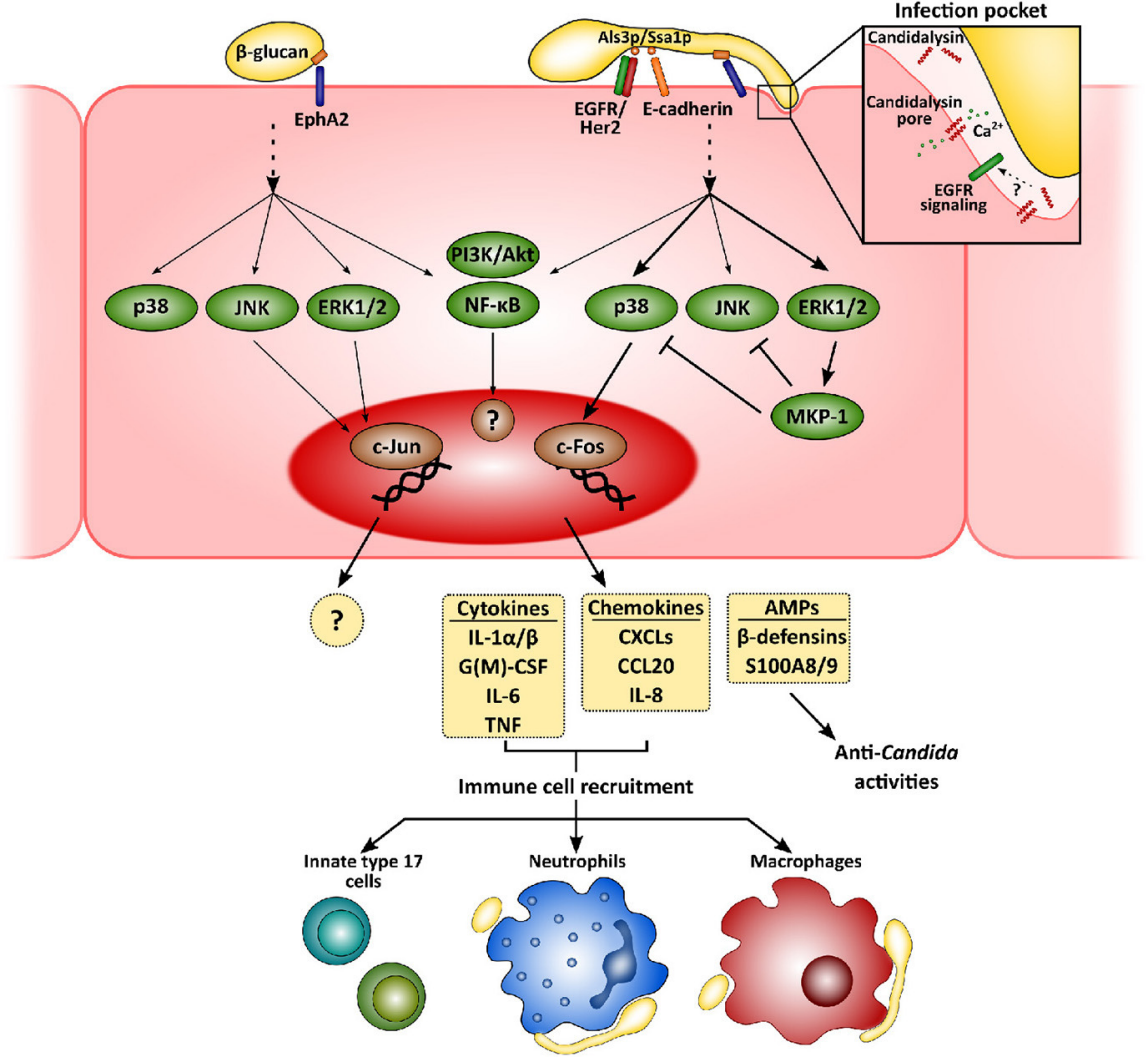
Mucosal innate immunity to *Candida albicans*. Recognition of *C. albicans* by epithelial cells is mainly mediated by the β-glucan receptor Eph2A. This leads to the activation of epithelial cells that greatly depends on fungal morphology and secretion of the hyphal toxin candidalysin. While yeast cells activate PI3K/Akt and NF-κB, with a weaker activation of p38, JNK, and ERK1/2 MAPK signaling pathways, hyphal growth and the release of candidalysin to the infection pocket promote a sustained, strong activation of all three MAPK pathways. The first leads to the recruitment of c-Jun, with as-yet unknown transcriptional consequences. Hyphal activation, however, induces c-Fos activation, leading to the release of pro-inflammatory molecules and antimicrobial peptides. These molecules will eventually help to clear fungal invasion and recruit more immune cells to the infection foci, such as neutrophils, macrophages and innate Th17 cells.

Mucosal immunity to *Candida* has extensively been reviewed recently, including its recognition by and interactions with epithelial cells (Naglik et al., 2017; Richardson et al., 2018; Nikou et al., 2019; Swidergall, 2019), and the role of innate immune cells (Verma et al., 2017a; Richardson et al., 2019). Therefore, we will give a brief general overview of the current view of anti-*Candida* immunity at the mucosae (Figure 1).

Physical contact and adherence of *C. albicans* to epithelial cells is essential for the interaction with a host. This process is mediated by fungal adhesins, such as the agglutinin-like sequence (Als) protein family, interacting with different host receptors facilitating fungal cell attachment [e.g., epidermal growth factor receptor (EGFR) and E-cadherin] (Moyes et al., 2015; Nikou et al., 2019). Once *C. albicans* has adhered, epithelial cells detect pathogen-associated molecular patterns (PAMPs; e.g., β-glucans or mannans) and other fungal markers resulting in activation of host cell responses. Although host sensing of *C. albicans* has been studied to a greater or lesser extent for both epithelial and immune cells, it is still not completely understood (Swidergall, 2019). Several types of pattern recognition receptors (PRRs) mediate *Candida* recognition by innate immune cells, including Toll-like or C-type lectin receptors, but their relevance at the mucosa may well be different to the systemic environment (Swidergall, 2019). In fact, while dectin-1 is critical during systemic infections (Taylor et al., 2007), its role during oropharyngeal candidiasis is minor (Verma et al., 2017b) and its expression can even be downregulated in oral epithelial cells when challenged with the fungus (Moyes et al., 2014). Interestingly, non-classical receptors including E-cadherin and EGFR/Her2 have been implicated in the epithelial recognition process (Phan et al., 2007; Sun et al., 2010; Zhu et al., 2012). More recently, EphA2 has been described as a β-glucan recognition receptor mediating immune responses by both oral epithelial cells (Swidergall et al., 2018) and neutrophils (Swidergall et al., 2019b), where it boosts Fcγ mediated antifungal responses such as reactive oxygen species (ROS) generation, and type 17 immunity. In addition, whilst the yeast form is the most immunogenic in the systemic environment, hyphae are the activating morphotype for mucosal surfaces (Moyes et al., 2010; Cheng et al., 2011).

Subsequent to adhesion to the epithelial surface, *Candida* undergoes a transition to hyphal growth that is then associated with the following invasion of the epithelium. This invasion occurs via two different mechanisms—induced endocytosis and active penetration. While the first is induced by the engagement of the *C. albicans* proteins Ssa1p and Als3p to cadherins and EGFR/Her2 (Phan et al., 2007; Sun et al., 2010; Zhu et al., 2012), active penetration occurs when the growing hyphal tip pushes epithelial cell membrane, eventually leading to cell damage. Notably, during the first of these processes *C. albicans* forms an “invasion pocket” (Zakikhany et al., 2007; Wächtler et al., 2012) in the epithelial cell into which the fungus presumably secretes and accumulates a cytotoxic peptide, candidalysin (Moyes et al., 2016). This cytolysin has a dual effect, involved in both the induction of host cell damage and the development of immune responses by epithelial cells (Moyes et al., 2016; Naglik et al., 2019). Although EphA2 mediates *C. albicans* β-glucan recognition, secondary stimuli through different signaling circuits are still needed for the development of a proper immune response (Swidergall et al., 2018). In fact, candidalysin seems to be indispensable for driving pro-inflammatory outcomes in epithelial (Moyes et al., 2016; Richardson et al., 2017) and endothelial cells (Swidergall et al., 2019a). Recently, a signaling circuit involving EGFR has been identified as critical for the induction of immune responses by candidalysin in epithelial cells (Ho et al., 2019). Importantly, EGFR is not a direct receptor for this cytolysin but a key component of the signaling pathways associated with candidalysin-mediated induction of immune responses by mechanisms including intracellular calcium signaling and EGFR endogenous ligands, such as epiregulin and epigen (Ho et al., 2019). Therefore, this novel circuit does represent new opportunities for the development of novel therapeutic strategies.

Recognition of *C. albicans* promotes intracellular events in epithelial cells that largely depend on fungal morphology and burden (Moyes et al., 2010, 2011), which have in turn been related to the different *C. albicans*-host relationships, namely commensalism and pathogenesis (Tang et al., 2016). While commensal yeasts or low hyphal burdens promote an activation of PI3K/Akt and NF-κB, with weaker, transient activation of p38, JNK, and ERK1/2 MAPK signaling pathways, detection of high levels of *C. albicans* hyphae results in the strong, sustained activation of all three MAPK pathways. This latter leads to activation of c-Fos within the AP-1 transcription factor and eventually, to the secretion of an array of antimicrobial peptides, alarmins, and pro-inflammatory cytokines (Moyes et al., 2010, 2014; Verma et al., 2017b, 2018; Nikou et al., 2019).

Release of these pro-inflammatory factors lead to the recruitment of immune cells to the infection foci, mainly neutrophils and macrophages (Netea et al., 2008; Erwig and Gow, 2016). Neutrophils are considered the most relevant innate immune cells to control mucosal fungal infections in the oral cavity (Cheng et al., 2012), providing epithelial protection in both contact-dependent and -independent mechanisms (Schaller, 2004; Weindl et al., 2007). However, their role in vulvovaginal candidiasis is more restricted due to a mechanism that, at least in animal models, involved the inhibition of complement receptor 3/Mac-1 by the proteoglycan heparan sulfate (Yano et al., 2017a,b). *Candida albicans* induces rapid transcriptomic changes in neutrophils, activating the expression of immune pathways, and the rearrangement of the cytoskeleton and vesicular trafficking (Niemiec et al., 2017). This leads to the phagocytosis of *C. albicans* yeasts and germlings that may be killed in the phagolysosome (Cheng et al., 2012). However, when neutrophils detect higher microbial size (i.e., long hyphae) (Branzk et al., 2014; Warnatsch et al., 2017), they undergo a specialized form of cell death (NETosis) and form neutrophil extracellular traps (NETs). Interestingly, *C. albicans* can evade the effect of NETs by releasing DNAse to the extracellular medium (Zhang et al., 2017) or by forming biofilms (Kernien et al., 2017), which renders fungal cells less accessible to immune cells.

*C. albicans* also induces monocyte differentiation and polarization to pro-inflammatory macrophages (Leonhardt et al., 2018), regulating their transcriptional landscape (Muñoz et al., 2019), microRNA expression (Croston et al., 2018) and extracellular vesicle release (Reales-Calderón et al., 2017). While macrophages also efficiently phagocytize *Candida*, their killing ability is lower than that of neutrophils, leading to the smaller contribution of macrophages during *C. albicans* infections (Cheng et al., 2012). Moreover, the fungus can induce macrophage cell death by hypha-mediated membrane piercing (Vylkova and Lorenz, 2014; Westman et al., 2018) or activation of NLRP3-dependent pyroptosis (Uwamahoro et al., 2014; Wellington et al., 2014; Vylkova and Lorenz, 2017). The pyroptotic process seems to be independent from candidalysin, although the toxin is responsible for the activation of NLRP3 inflammasome and caspase-1 in macrophages and dendritic cells (Kasper et al., 2018; Rogiers et al., 2019).

In recent years, other sets of innate immune cells have attracted the attention of several research groups due to the essential role of IL-17-mediated responses for antifungal immunity. Notably, these responses are crucial for anti-*Candida* immunity in oral mucosal tissues (Gaffen et al., 2011; Conti and Gaffen, 2015; Li et al., 2018; Gaffen and Moutsopoulos, 2020). In the oropharyngeal candidiasis (OPC) mouse model, mice deficient in IL-17RA, IL-17RC, or IL-23p19 show increased susceptibility to oral candidiasis (Conti et al., 2009; Ho et al., 2010), independently on the *C. albicans* strain used (Schönherr et al., 2017). More importantly, human individuals bearing inborn mutations in genes coding for IL-17 pathway components, such as CARD9 or STAT3, show greatly increased morbidity of these infections. In sharp contrast, IL-17-mediated responses are dispensable for vulvovaginal candidiasis (Yano et al., 2012; Peters et al., 2019). This is in accordance with previous observations in HIV positive patients who show increased oral thrush prevalence, but no vulvovaginal or systemic candidiasis, highlighting the site-specificity of these responses to the oral cavity (Fidel, 2011).

Among the six cytokines composing the IL-17 family (IL-17A, F, B, C, D, and E), oral epithelial cells only express IL-17C (Monin and Gaffen, 2018). However, deletion of genes coding for IL-17C or one of its receptor subunits (IL-17RE) does not lead to worse outcomes during systemic or oral candidiasis (Conti et al., 2015; Trautwein-Weidner et al., 2015). Despite this, epithelial cells do express IL-17A receptor and its engagement induces a wide range of responses in the oral epithelium, including AMP production, and cytokine and chemokine release (Conti and Gaffen, 2015). Moreover, its conditional deletion in oral epithelial cells leads to similar susceptibility to OPC as complete IL-17RA knockout mice strains (Conti et al., 2016). The identification of the most relevant sources of IL-17 during oral candidiasis in OPC is still under debate (Cua and Tato, 2010; Conti and Gaffen, 2015). While the main origin seems to be “natural” Th17 cells (IL-17^+^TCRαβ^+^, nTh17) present in the oral mucosa, the role of γδ-T cells and Innate Lymphoid Cells type 3 (ILC3) has been controversial and needs to be further confirmed (Gladiator et al., 2013; Conti et al., 2014; Sparber et al., 2018). Notably, candidalysin release by *C. albicans* hyphae during oral epithelium colonization seems to make a major contribution to triggering IL-17 production and nTh17 cell expansion, and acts synergistically with IL-17 to enhance epithelial cytokine and chemokine production (Verma et al., 2017b).

However, in contrast to healthy laboratory mice, humans usually harbor *C. albicans* as part of their mycobiota. Therefore, these animals usually experience their first contact with the fungus during OPC experiments, and consequently, most responses are of innate origin (Verma et al., 2017b). In fact, recall infection experiments in mice showed a robust antigen-specific response mediated by IL-17-producing CD4^+^ T cells (Hernández-Santos et al., 2013), and *C. albicans* is the major fungal inducer of human Th17 responses (Bacher et al., 2019). Thus, since currently there are no markers to discriminate native and conventional Th17 cells, their precise role in the IL-17 release during mucosal candidiasis in humans is not completely known (Gaffen and Moutsopoulos, 2020).

## Relevance of Fungal Factors for the Interaction With the Host: the Janus-Faced *C. albicans*

As part of the human mycobiota, *C. albicans* has evolved mechanisms that allow the fungus to thrive in numerous environmental conditions, which overall means no harm to its host. However, with some environmental changes, *C. albicans* can take advantage and overgrow, thereby colonizing new niches inside the host in a process that usually leads to disease. Thus, *C. albicans* is a pathobiont with a high degree of plasticity at the cellular, metabolic and molecular levels.

### *Candida albicans* as the Major Fungal Pathogen

The ability of *C. albicans* to switch between different morphologies has been the focus of a great number of studies, especially the yeast-to-hypha transition and its association with fungal pathogenesis (Jacobsen et al., 2012). Importantly, recognition of *C. albicans* by immune cells is strongly influenced by its morphology due to differential PAMP exposure on the different morphologies, among other factors (Gow et al., 2012). In addition, during invasive hyphal growth *C. albicans* induces the expression of a wide array of genes that contribute to invasion of host tissues and evasion of immune response, including the previously mentioned proteinases and *ECE1*, the precursor of candidalysin (Nantel et al., 2002).

As with most fungal species, *C. albicans* is able to secrete a wide repertoire of proteins that help the fungus thrive in different environments, including the mammalian host (Klis and Brul, 2015). The complex secretome of *C. albicans* includes proteins secreted freely and proteins contained in extracellular vesicles (Gil-Bona et al., 2018), the latter promoting activation and cytokine release by innate immune cells (Vargas et al., 2015). Interestingly, functional analysis of these secreted proteins identifies several enzymes involved in central metabolism pathways (e.g., Eno1p, Tdh3p or Adh1p) or stress response proteins (as Hsp70p). The role of these enzymes in host-microbe interactions is controversial and they are often referred to as moonlighting proteins due to their multiple functions (Karkowska-Kuleta and Kozik, 2014; Gancedo et al., 2016). For example, the molecular chaperone Ssa1p can also act as a surface adhesin, co-operating with the adhesin Als3p to mediate *C. albicans* attachment to epithelial cells (Phan et al., 2007; Sun et al., 2010; Zhu et al., 2012).

Additionally, many other secreted elements are actively involved in *C. albicans* pathogenesis, including hydrolytic enzymes and the recently described toxin candidalysin. Among the hydrolytic enzymes, secreted aspartyl proteinases (SAPs) are the most well-known (Naglik et al., 2003; Rapala-Kozik et al., 2018). This group of enzymes is formed of ten members that are either secreted (Sap1-8p) or kept anchored to the cell wall (Sap9-10p) (Albrecht et al., 2006). Notably, they degrade relevant components of the immune response in the mucosae, as cadherins, complement system molecules or immunoglobulins (Naglik et al., 2003; Rapala-Kozik et al., 2018). However, while these proteins seem to help *C. albicans* to evade the immune responses, their relation to epithelial damage and mucosal invasion is less clear. Although the use of the SAP inhibitor pepstatin A showed a decrease in epithelial cell damage (Schaller et al., 1999; Naglik et al., 2008), other authors suggested they are dispensable for this process (Lermann and Morschhäuser, 2008). In addition to impacts on the host, SAPs play an important role in direct fungal virulence. Cleavage action of SAPs on the *Candida* mucin-like protein Msb2p promotes MAPK signaling in the fungus (Román et al., 2009; Saraswat et al., 2016), whilst *msb2*Δ*/*Δ null mutants show defects in biofilm formation and virulence capacity during OPC (Puri et al., 2012). Remarkably, the highly glycosylated protein fragment shed from Msb2p after proteinase action (Msb2^*^) confers protection to *C. albicans* from the antimicrobial peptides LL-37 and histatin-5 (Szafranski-Schneider et al., 2012; Swidergall et al., 2013), indicating that this protein also has multiple roles in virulence and host interactions.

Secretions by *C. albicans* also contain a great number of lipases (Lip1-10p) and phospholipases (belonging to four classes, PLA-D, although only PLB and PLC are secreted) (Schaller et al., 2005; Nikou et al., 2019). Phospholipase activity has long been associated with yeast adhesion to epithelial cells and mortality in mice (Barrett-Bee et al., 1985). Expression of these proteins differs between *in vivo* systemic and orogastric infections, and samples from patients suffering from oral candidiasis (Stehr et al., 2004; Schofield et al., 2005). However, their precise role during epithelium infection is yet to be defined.

### *Candida albicans* as an Essential Part of the Healthy Mycobiota

*Candida* is the most abundant fungal genus in healthy oral and gut mycobiota, being both commensal and pathogenic depending on the environmental conditions (Witherden et al., 2017). Although significant work has been carried out over the years investigating host and fungal responses and functions during infection, little is known about the functions and biology of these species when they are part of the commensal microbiota. Notably, analysis of *C. albicans* commensalism in animal models is quite difficult, as healthy wildtype mice under clean laboratory conditions do not carry this fungus as part of their normal microbiota and are therefore, immunologically naïve to it (Conti et al., 2009). In spite of this, mice resist gastrointestinal (GI) tract colonization by most *C. albicans* strains by mechanisms involving the induction of HIF1α and LL-37 in host cells by members of the gut microbiota, such as clostridial Firmicutes and Bacteroidetes (Fan et al., 2015; Mishra and Koh, 2018). Thus, most colonization models are based on mice exposed to antibiotic therapies to induce gut dysbiosis or with an altered immune system (Prieto et al., 2016).

Although *C. albicans* morphological plasticity has been mainly studied in the context of pathogenesis (Gow et al., 2012), regulation of morphology during commensal lifestyle seems to be vital (Noble et al., 2017; Pérez, 2019). In fact, the GUT (gastrointestinal induced transition) morphotype was described after inducing Wor1p overexpression by sequential passages through the mouse GI tract. Notably, this phenotype shows improved fitness in the GI tract and a specific transcriptional profile (Pande et al., 2013). More recently, Tso et al. produced gut-evolved strains by inducing genetic rearrangements after 8–10 weekly passages through the gut of antibiotically-treated mice. Many of these “evolved” strains lost their filamentation ability by accumulating loss-of-function mutations in *FLO8* (Tso et al., 2018), an essential transcription factor for yeast-to-hypha transition (Cao et al., 2006). Importantly, mutation of this gene or loss of yeast-hyphal transition is not enough to explain this entire phenomenon, as these “evolved” strains showed a more extreme adaptation than the *flo8*Δ*/*Δ mutant, indicating the potential importance of other intraspecific genetic variations in host-fungal interactions. Although other factors may be involved, yeast-hyphal transition still seems to be highly relevant for fungal colonization of the GI tract as yeast-locked mutants (*efh1*Δ*/*Δ, *efg1/cph1*Δ*/*Δ) show increased persistence in comparison to wild type strains, while hypha-locked ones (*nrg1*Δ*/*Δ) exhibit decreased colonization capacity (White et al., 2007; Pierce and Kumamoto, 2012; Pande et al., 2013; Vautier et al., 2015). While expression of the morphological transition regulators *EFH1* (White et al., 2007) and *EFG1* (Pierce and Kumamoto, 2012), or even *EFG1* gene dosage (Liang et al., 2019), seems to have a role during initial steps of GI colonization, their presence limits long-term colonization as observed when using null strains. However, the relevance of the morphological transition is still under debate, as several reports have shown that the presence of yeasts and hyphae during the commensal colonization in the gut is either similar (Witchley et al., 2019), or there are even more hyphae present (Böhm et al., 2017). Therefore, further studies should address the precise role of these and other genetic regulators to determine their relevance for commensalism and their differential presence/expression in beneficial and pathogenic strains.

In an effort to find other genetic regulators of the colonization capacity of *C. albicans* in the GI tract, a screen of more than 70 fungal transcription factors null strains identified six mutant strains with impaired GI tract colonization, including Tye7p, Hms1p, Rtg1p, Rtg3p, Lys144p, and orf19.3625 (Pérez et al., 2013). Remarkably, most of them regulate *C. albicans* central metabolism (Tye7p, glycolysis; Rtg1/3p; galactose catabolism; Lys144p, lysine biosynthesis), which has previously been shown to be upregulated during growth in the mouse cecum compared to laboratory conditions (Rosenbach et al., 2010). More recently, three new transcriptional regulators (as well as the well-described Efg1p) where null mutant strains showed increased fitness in the mouse GI tract (Witchley et al., 2019). The genes identified in this study, *RBG1, ROB1, TEC1*, and *EFG1*, are transcription factors regulating yeast-to-hypha transition and virulence factor expression, and all indirectly induce hyphal formation through Ume6p, a “master regulator” controlling filamentation regulatory network (Banerjee et al., 2008; Witchley et al., 2019). Interestingly, wild type and *ume6*Δ*/*Δ show similar filamentation rates in the gut, showing that morphology switch *per se* is not essential. Importantly, the virulence factors Sap6p and Hyr1p were found to be essential for Ume6p-related inhibition of GI tract colonization (Witchley et al., 2019). As well as these effects in yeast-hyphal transition, fungal MAPK signaling has also been associated with *C. albicans* gut colonization (Román et al., 2019), not just with virulence (Román et al., 2007). In a recent study, all three MAPK pathways (Mkc1p, Cek1p, and Hog1p) were identified as important for fungal fitness in the gut, but in particular the HOG pathway as null mutants in *HOG1* or *PBS2* were unable to colonize the GI tract (Prieto et al., 2014).

Although oral epithelial cells are able to discriminate between colonizing or invading *C. albicans* and, consequently, respond by triggering weaker or stronger inflammatory responses, respectively (Moyes et al., 2010, 2011; Tang et al., 2016), little is known about the fungal factors involved in this process. Although the fungal virulence factor, candidalysin, has been shown to be critical to identifying the presence of invading hyphae (Moyes et al., 2016), the factors involved in recognition and interaction with colonizing yeast cells are currently poorly understood. At the systemic level, monocytes and neutrophils respond strongly to yeast cells, phagocytosing and killing them, at mucosal surfaces this morphotype does not generate strong responses (Moyes et al., 2010; Cheng et al., 2011). Similar to the increased fitness of GUT morphology in the GI tract, the yeast-like gray cell morphotype appears to facilitate colonization of the tongue (Tao et al., 2014). Persistence in the oral mucosa largely depends on the fungal strain. While persistent fungal isolates exhibit similar burdens to those showed by highly infective strains in an OPC mouse model (e.g., the hypervirulent clinical isolate SC5314), they induced neither loss of weight in infected animals nor promoted acute inflammation. Despite this, neither strain origin nor filamentation *in vitro* seem to be good predictors of pathogenicity *in vivo*, while damage and IL-1α induction in epithelial cells resembled virulence at the oral mucosa (Schönherr et al., 2017). In addition, selective pressure in the oral cavity during OPC may promote rapid chromosomal reorganization in *C. albicans* (Forche et al., 2018), leading to a more “commensal-like” phenotype and exhibiting decreased virulence and pro-inflammatory features (Forche et al., 2019). Recently, using the same strategy as for GI colonization (Pérez et al., 2013), Meir and colleagues analyzed the role of ~70 selected *C. albicans* transcription factors during OPC, and identified that deletion of *CUP9* and *ZCF8* increased persistence, while *ZCF21, ZCF27*, ORF19.4649, and ORF19.217 null mutant strains displayed reduced survival in the oral cavity. Surprisingly however, a more in-depth analysis of *cup9*Δ*/*Δ infection *in vivo* showed a higher filamentation ability and more colonization foci in the tongue than the wild-type strain without eliciting increased immune response or cell damage (Meir et al., 2018), suggesting that other factors, together with filamentation, are relevant. Notably, recent studies have shown that intraspecific differences not associated with fungal morphology between *C. albicans* isolates lead to diverse recognition/immune responses by the oral epithelium (Kirchner et al., 2019), although virtually all strains induce conserved IL-17-mediated responses (Schönherr et al., 2017). Thus, this highlights the importance of other fungal factors that contribute to the balance between commensalism and pathogenesis in the oral cavity (Swidergall, 2019).

## New Insights in Immunity to Mucocutaneous Candidiasis

**Figure 2:**
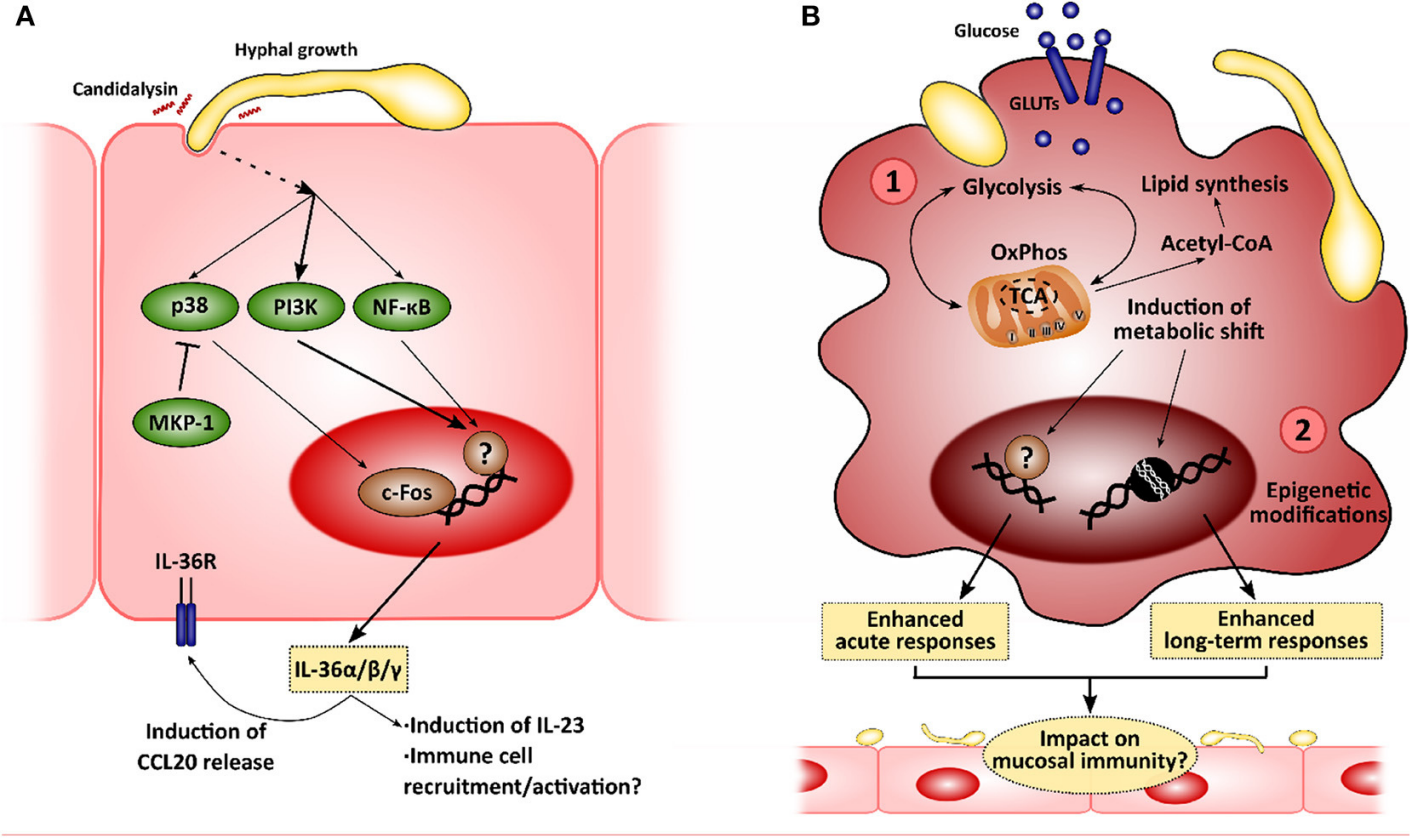
New insights in the mucosal immunity to *Candida albicans*. Novel approaches to analyze the interactions between epithelial cells and *C. albicans* will allow to identify new intervention points to treat mucocutaneous infections. **(A)** Recently, *C. albicans* was described to induce IL-36 release in epithelial cells in a morphology- and candidalysin-dependent manner, and IL-36 signaling has been observed to be important for proper fungal clearance during oropharyngeal candidiasis. **(B)** *C. albicans* has also been shown to induce metabolic reprogramming in innate immune cells, such as macrophages, which is linked to the modulation of immune responses (1). This metabolic shift has been associated with the epigenetic remodeling in these immune cells, leading to the development of long-term responses, or innate immune training (2). The impact of these mechanisms in the epithelial immune responses to *C. albicans* is yet to be elucidated.

In recent years, several factors and mechanisms related to innate immune responses have been described and shown to be critical during microbe-host interactions. These include the importance of poorly studied cytokine families, such as the IL-36 family, and the impact of long-term immunity (i.e., innate immune memory) and the control of immune responses by cellular metabolism (Figure 2). While their importance in the context of anti-*Candida* mucosal immunity is yet to be explored extensively, we hypothesize that they may be of outstanding relevance as additional mechanisms to control fungi in both health and disease.

### Role of IL-36 Cytokines in Anti-*Candida* Responses

The candidalysin-dependent induction of IL-1α/β expression in epithelial cells (Moyes et al., 2016) and during acute OPC (Verma et al., 2017b) is essential for host defense against *C. albicans*. Importantly, IL-1 signaling promotes IL-17-driven responses (Conti et al., 2009; Verma et al., 2017b), and its defective performance leads to reduced cytokine/chemokine release, impaired neutrophil recruitment and consequently, increased fungal burdens (Altmeier et al., 2016).

Included in the IL-1 cytokine superfamily, IL-36 members are emerging as important mediators of the immune response to microbial infections and inflammatory skin diseases (Jensen, 2017; Buhl and Wenzel, 2019). IL-36 family includes three agonistic cytokines, IL-36α, IL-36β, and IL-36γ, and one antagonistic molecule, IL-36RA, all of them signaling through the dimer composed of IL-36R and IL-1R accessory protein. This cytokine family is involved in the activation of myeloid cells (including monocytes and dendritic cells) and epithelial cells, such as skin keratinocytes, contributing to leukocyte recruitment or AMP secretion (Gresnigt and van de Veerdonk, 2013; Jensen, 2017; Bassoy et al., 2018; Swindell et al., 2018).

However, little is known on the role of IL-36 in mucosal tissues, although an increasing number of studies have shown the relevance of these proteins for the response to a wide variety of stimuli (Jensen, 2017). Importantly, IL-36γ, IL-23, and IL-22 are induced during intestinal mucosa damage, and are essential for tissue repair, host cell survival, and antimicrobial activity (Ngo et al., 2018). Vaginal epithelial cells react to diverse microbial products by triggering *IL36G* gene expression, which subsequently enhances the expression, transcription and release of AMPs and other pro-inflammatory mediators, including IL-36γ itself (Winkle et al., 2016). Additionally, IL-36 has been shown to be important in a number of infections at different mucosal sites. At these surfaces, it induces a series of different AMPs and other protective factors, including peptidoglycan recognition protein 2, human β-defensin 2, or matrix metallopeptidase 9 (Huynh et al., 2016; Aoyagi et al., 2017; Kovach et al., 2017; Nanjo et al., 2018; Scholz et al., 2018; Heath et al., 2019).

Unsurprisingly, *C. albicans* joins the list of microbes that induce the expression of all IL-36α/β/γ, showing an increase in production in a mouse model of OPC and in human oral epithelial cells. This increase is hypha-dependent and is mediated by candidalysin, as recently shown by using toxin-deficient strains and the peptide itself (Verma et al., 2018). More importantly, defective/absent IL-36 signaling results in decreased body weight and increased fungal burdens in the tongue. However, while IL-1R^−/−^ mice show defective IL-17 expression and IL-17-driven responses with normal IL-23 expression, IL-36R^−/−^ mice show decreased IL-23 expression with no effect on IL-17 and subsequent gene expression. This may be related to worsened OPC outcomes. In fact, IL-23-deficient mice exhibit higher fungal burdens, comparable to those observed in Act1^−/−^ (showing defective IL-17 signaling), and whilst this may be part due to a reduced type 17 response in these animals (Sparber et al., 2018), there is evidence available to suggest that IL-23 has IL-17-independent effects (Lee et al., 2015; Maxwell et al., 2015; Verma et al., 2018). These observations highlight the role of IL-36-mediated protection as an independent pathway contributing to immune mucosal protection to *C. albicans* infections (Verma et al., 2018).

### Role of Innate Immune Memory in Mucosal Surfaces Defense

The innate immune system has critical roles in antifungal immunity both at systemic and mucosal levels, as previously discussed. Traditionally, innate immune cells have been considered to develop non-specific and “simple” mechanisms to eradicate pathogen invasion based on the recognition of PAMPs but lacking any “memory” of pathogens. This contrasts with the ability of the adaptive arm of the immune system to “remember” pathogens and orchestrate more specific and efficient responses (e.g., antibodies). However, this classical view of the immune system assigning differential specificity and the ability to develop “memory” states to innate and adaptive cells has been recently challenged.

Cells of the innate immune system have now been shown to undergo long-term changes associated with previous encounters with PAMPs, either in isolation (e.g., LPS, β-glucan) or in complex mixtures (e.g., the mycobacterial vaccine BCG, heat-killed microbes), that result in subsequently modulated responses (Netea and van der Meer, 2017). Collectively, these responses have been termed innate immune memory, and are currently divided into two general outcomes: Trained Innate Immunity, and Tolerance (Boraschi and Italiani, 2018), the difference mainly being the capacity of experienced cells to induce an enhanced or decreased production of inflammatory effectors (e.g., TNF), respectively, upon a secondary encounter usually with a distinct stimulus.

While LPS-induced tolerance had already been described both *in vitro* and in clinical settings (West and Heagy, 2002; Seeley and Ghosh, 2017), leading patients suffering from septicemia to the so-called state of “immune-paralysis” (Hotchkiss et al., 2013), trained immunity was described more recently. Quintin et al. firstly observed an increased T cell- and B cell-independent protection to *C. albicans* systemic infection after pre-exposing mice to a non-lethal fungal dose (Quintin et al., 2012). Further analyses showed how β-glucan exposure functionally reprograms monocytes through Dectin-1/Raf-1/NF-κB pathways, with these undergoing epigenetic modifications leading to enhanced long-term responses (Cheng et al., 2014; Saeed et al., 2014; Netea et al., 2016; van der Heijden et al., 2018). In addition, a metabolic shift coordinated via Akt/mTOR/HIF1α is induced in trained cells toward aerobic glycolysis, which presumably boosts the enhanced inflammatory response (Arts et al., 2016). In contrast, LPS-tolerized cells display decreased oxidative phosphorylation (OxPhos) and glycolytic rates (Cheng et al., 2016). More recently, Tso et al. showed that gut-evolved *C. albicans* strains induced the “trained” phenotype with even greater efficiency than the wild-type SC5314 strain (Tso et al., 2018), suggesting that induction of trained immunity is based on more complex mechanisms and plays a critical role in host-microbiome homeostatic responses.

Although these innate immune memory mechanisms have mainly been described in monocytes/macrophages and other immune cell types (reviewed in Netea et al., 2016), new studies are beginning to show that non-immune cells are also able to develop these memory-like responses (Hamada et al., 2019), including mesenchymal stem cells (Liu et al., 2016), hematopoietic progenitors (Kaufmann et al., 2018; Mitroulis et al., 2018) and skin epithelial stem cells (Naik et al., 2017). Concerning epithelial cells at mucosal surfaces, there is significant evidence for a priming event driven in a morphology-independent fashion. Initial investigations identifying the role of the Akt/mTOR pathway in oral epithelial cell responses to *C. albicans* showed that this pathway is morphology independent, and that inhibition of this pathway resulted in an increase in damage inflicted by the fungus, as well as a decrease in the cytokine response, suggesting a morphology-independent priming effect on subsequent responses to hyphae and candidalysin toxin effects (Moyes et al., 2014). As above mentioned, human oral and vaginal epithelial cells have grown mechanisms to detect fungal burden and discriminate between commensal and pathogenic states of *C. albicans* (Moyes et al., 2010, 2011; Tang et al., 2016), suggesting that long-term responses may regulate host-*C. albicans* interactions both in health and disease. More recently, Alburquenque et al. showed that short pre-stimulation of reconstituted human epithelium with *C. albicans* heat-killed yeast cells increased human β-defensin 3 expression, and reduced fungal viability and adherence (Alburquenque et al., 2019).

Whether these phenomena are genuine long-term “training” events or simple priming events remains to be elucidated. However, their discovery would be of outstanding relevance for the development of novel therapies, and even long-awaited vaccines, to deal with mucocutaneous candidiasis (Cassone, 2018), and thus represent one of the most significant questions in need of answering.

### Role of Cell Metabolism During Host-*C. albicans* Interactions

It has become increasingly evident over the past decades that immune cells rely on changes in cellular metabolism to mount effective antimicrobial responses, with glucose metabolism being a central player in immune cell function (Pearce and Pearce, 2013). As a result of the recognition of microbial ligands, macrophages upregulate glucose uptake and its anaerobic catabolism instead of relying on the tricarboxylic acid (TCA) cycle and mitochondrial OxPhos (use of glycolysis coupled to lactic acid fermentation in normoxic conditions, i.e., aerobic glycolysis). Thus, they obtain the required energy for boosting antimicrobial mechanisms and cytokine production (El Kasmi and Stenmark, 2015; Kelly and O'Neill, 2015). This behavior resembles the so-called Warburg effect observed in cancer cells, which predominantly use aerobic glycolysis (Warburg et al., 1927). Notably, this metabolic shift (and the consequent responses) varies in intensity and nature depending on the microbial insult used or the PRR involved in the process (Lachmandas et al., 2017), indicating degree of selectivity.

*C. albicans* has been shown to induce this metabolic reprogramming in monocytes/macrophages (Traven and Naderer, 2019), although depends on fungal morphology. While both heat-killed yeast and hyphae induce the expression of genes involved in glycolysis and glutaminolysis, hyphae-stimulated cells respond to a lesser extent (Domínguez-Andrés et al., 2017). Interestingly, activation using *C. albicans* yeasts led to an increase in cellular respiration (an indicator of OxPhos activity), contrasting with the profile exhibited by LPS/IFNγ-activated macrophages (El Kasmi and Stenmark, 2015). Blocking glycolysis at different intervention points induces defective pro-inflammatory cytokine release and ROS species production, and worse outcomes during *in vivo* systemic infections (Domínguez-Andrés et al., 2017). Similarly, *C. albicans* induce NK cells to change their metabolism toward glycolysis, which is required for proper immune and cytolytic responses of this cell type (Hellwig et al., 2016).

As previously mentioned, the metabolic profile of cancer cells is switched toward anaerobic glycolysis to support their rapid proliferation and survival, and precancerous lesions in the gut mucosa also exhibit increased markers of this metabolic reprogramming (Cruz Dela et al., 2017). Thus, *C. albicans*-induced metabolic shift could be linked to a more pathogenic state, which may include excessive inflammation and even, oncogenesis. Several studies have shown the association of *C. albicans* with cancer (Chung et al., 2017; Perera et al., 2017), and the ability of this fungus to produce potent carcinogens such as nitrosamines or acetaldehyde that induce DNA adducts and lead to point mutations (Ramirez-Garcia et al., 2014). In addition, expression of inflammatory and adhesion molecules in endothelial cells induced by the presence of the fungus has been linked to metastasis promotion in the liver (Ramirez-Garcia et al., 2011, 2013).

At mucosal surfaces, epithelial cells can also undergo metabolic reprogramming in response to microbes. In the case of bacteria, *Citrobacter rodentium* has been shown to repress carbohydrate metabolism in mouse intestinal epithelial cells (Hopkins et al., 2019), whilst *Staphylococcus aureus* promotes glycolytic activity in skin keratinocytes (Wickersham et al., 2017). More importantly, catabolic pathways are upregulated during an OPC mouse model, with increased expression of relevant metabolic regulators (e.g., HIF1α) in tongue epithelial cells after 1 day of infection. This metabolic reprogramming is coupled with the characteristic enhanced immune responses of the model, which highlights the relevance of this shift in metabolism for the infection outcome (Kirchner et al., 2019). However, further analyses of the implications of host cell metabolism during mucosal candidiasis must be performed in order to completely understand which metabolic pathways are involved in mucosal anti-*Candida* responses. As previously discussed, development of immune memory is greatly influenced by a change in cell metabolic profile of innate immune cells and therefore, may also be of outstanding relevance for long-term responses of epithelial cells to *C. albicans*.

In these scenarios, it is also important to recognize that the host cell is not operating in an enclosed environment—at the very least, the microbe is also present with its own metabolic programs. Notably, *C. albicans* can act as both extracellular and intracellular pathogen and has the ability to regulate its metabolism to adapt to highly different environments (Brown et al., 2014; Miramón and Lorenz, 2017). Regulation of *C. albicans* carbohydrate metabolism is highly complex, and transcription factors involved in metabolic regulation, such as Tye7p, play a key role in virulence (Askew et al., 2009) and colonization (Pérez et al., 2013). While inside phagolysosomes in macrophages (Muñoz et al., 2019) or neutrophils (Niemiec et al., 2017), the fungus uses non-glucose carbon sources by activating fatty acid oxidation or glyoxylate pathway, with the latter being essential for fungal virulence and survival during infections (Lorenz and Fink, 2001). However, glucose is the preferred carbon source for *C. albicans* both during colonization and pathogenesis (Traven and Naderer, 2019), meaning that it competes with the host for it. In a recent study, Tucey et al. showed that *C. albicans* enhances its glycolytic activity through the transcriptional activators Tye7p and Gal4p after escaping from macrophages, until local glucose is depleted (Tucey et al., 2018). As previously stated, *C. albicans* activation of macrophages leads to them becoming overly dependent on glucose for use in aerobic glycolysis (Domínguez-Andrés et al., 2017; Tucey et al., 2018). The glucose depletion, together with this over-reliance of macrophages on glucose after their own metabolic reprogramming, increases immune cell death (Tucey et al., 2018). In contrast, *C. albicans* demonstrate a high degree of metabolic plasticity to continue proliferating, suggesting that the fungus exploits the macrophage immunometabolic reprogramming, turning it into a further virulence factor, to the detriment of host cells.

With the key role that metabolic reprogramming and subsequent pathways have in host and fungal responses during infection, targeting these pathways is an emerging therapeutic option for treating infections. Manipulating immune responses through therapeutic interventions of cellular metabolism has already been proposed not only for treating infections (Rao et al., 2019), but also for cancer or autoimmune diseases (Patel et al., 2019). As mentioned above, there is a competition between *C. albicans* and macrophages for locally available glucose. Therefore, one of the therapeutic options for improving host survival is to increase the availability of glucose and restoring host metabolic homeostasis to maintain macrophage function. In fact, increasing glucose in diet improved the outcome of systemic candidiasis by delaying macrophage death (Tucey et al., 2018). Regarding the therapeutic potential of manipulating fungal metabolism, metabolic adaptation in fungi, especially carbon metabolism, regulates virulence factor expression or recognition by immune cells, and therefore enzymes involved in these pathways could well be used as therapeutic targets (Brown et al., 2014). In fact, mutant strains or chemical interventions in glycolysis (Rodaki et al., 2006; Laurian et al., 2019) or fatty acid biosynthesis (Xu et al., 2009) showed defective growth, suggesting that these metabolic pathways can be important targets. Other therapeutic options that target *C. albicans* metabolism is to trigger endogenous nitric oxide (NO) production using compounds such as mdivi-1, which represses hyphal growth and the yeast-to-hypha transition and promotes metabolic reprogramming in the fungus (Koch et al., 2018). During growth and morphogenesis, *C. albicans* relies on OxPhos to produce ATP and therefore, mitochondria can be targeted for new antifungal drugs (Duvenage et al., 2019a). Importantly, inhibition of mitochondrial respiration not only reduces cell growth, but also induces changes in cell wall conformation. This leads to the unmasking of β-glucans on the *C. albicans* surface, thereby enhancing immune recognition and uptake by phagocytes. Conversely, Duvenage et al. also showed that pre-inhibition of respiration promotes the activation of virulence traits, leading to worse outcomes in systemic infection models (Duvenage et al., 2019b). However, which of these scenarios predominates at mucosal sites remains to be determined, and the scope for using such metabolic interventions for superficial infections is thus unclear.

## Conclusions

*C. albicans* has co-evolved with the human host establishing a complex relationship balanced between commensalism and pathogenicity. Although much work has been done investigating the classical immune responses of microbes, recent studies have begun to uncover the key role played by both epithelial cells and host and fungal metabolism in disease pathogenesis. Completely understanding of these relationships, will allow us to determine the precise roles *Candida* and other fungi have as commensals, but also to identify those factors that make possible the pathogenic fungal overgrowth. Exploring the role of emerging fields in microbiology and immunology, such as innate immune memory and the control of pathogenesis or immunity by cellular metabolism, will shed light to the still unknown events underlying anti-*C. albicans* immunity at mucosae.

## Author Contributions

All authors listed have made a substantial, direct and intellectual contribution to the work, and approved it for publication.

### Conflict of Interest

The authors declare that the research was conducted in the absence of any commercial or financial relationships that could be construed as a potential conflict of interest.
